# Clinical Trials in Older Adults with Cardiovascular Multimorbidity: A Perspective on Pragmatic Trial Design

**DOI:** 10.3390/jcm15124471

**Published:** 2026-06-09

**Authors:** Clara Bonanad, Didier Sánchez-Ospina, Sergio García-Blas, Gonzalo Luis Alonso-Salinas, Pedro Cepas-Guillén, Sergio Raposeiras-Roubín, Diego Segura-Rodríguez, Javier Pérez-Cervera, Carolina Ortiz-Cortés, Miguel Corbí-Pascual, Alicia Noguerón-García, Claudio M. Rivadulla-Varela, María Thiscal López-Lluva, David Vivas, Sofía Galant-Parrado, Sara Jordá-Climent, María Jesús Izquierdo-Ortiz

**Affiliations:** 1 Cardiology Department, University Clinical Hospital of Valencia, 46010 Valencia, Spain; sergiogarciablas@gmail.com (S.G.-B.); c.rivare@hotmail.com (C.M.R.-V.); 2 INCLIVA Biomedical Research Institute, 46010 Valencia, Spain; sgalant@incliva.es (S.G.-P.); sjorda@incliva.es (S.J.-C.); 3 Centro de Investigación Biomédica en Red de Enfermedades Cardiovasculares (CIBERCV), 46010 Valencia, Spain; 4 Clinical Analysis Department, Hospital Universitario de Burgos, 09006 Burgos, Spain; dsanchezo@saludcastillayleon.es; 5 Cardiology Department, Universidad Pública de Navarra (UPNA), Hospital Universitario de Navarra (HUN), Navarrabiomed, 31008 Pamplona, Spain; gonzalol.alonso@gmail.com; 6 Cardiology Department, Institut Clínic Cardiovascular, Hospital Clínic, Institut d’Investigacions Biomèdiques August Pi i Sunyer (IDIBAPS), 08036 Barcelona, Spain; pedro.cepasguillen@gmail.com; 7 Cardiology Department, University Hospital Álvaro Cunqueiro, 36312 Vigo, Spain; raposeiras26@hotmail.com; 8 Cardiology Department, San Cecilio University Hospital, 18012 Granada, Spain; diegoseguracardio@gmail.com; 9 Instituto de Investigación Biosanitaria ibs.GRANADA, 18012 Granada, Spain; 10 Acute Cardiac Care Unit, Cardiology Department, Complejo Hospitalario Universitario de Badajoz, 06006 Badajoz, Spain; jperezcervera@gmail.com; 11 Cardiology Department, Hospital Universitario Fundación de Alcorcón, 28922 Madrid, Spain; carol.ortizcortes@gmail.com; 12 Cardiology Department, Complejo Hospitalario Universitario de Albacete, 02006 Albacete, Spain; miguelcorbi@hotmail.com; 13 Hospital General Universitario de Albacete, 02006 Albacete, Spain; alicia_10nogueron@hotmail.com; 14 Complejo Hospitalario de León, 24080 León, Spain; mtl.lluva@secardiologia.es; 15 Hospital Clínico San Carlos, 28040 Madrid, Spain; dvivas@secardiologia.es; 16 Nephrology Department, Hospital Universitario de Burgos, 09006 Burgos, Spain

**Keywords:** geriatric cardiology, randomized controlled trials, multimorbidity, patient-centered outcomes, pragmatic clinical trials, COMGERCARDIO framework

## Abstract

Population aging has shifted the burden of cardiovascular disease toward older adults living with multimorbidity, frailty, cognitive vulnerability, functional impairment, and treatment burden. Yet much of the contemporary cardiovascular evidence still derives from randomized clinical trials (RCTs) designed around biologically homogeneous participants, limiting applicability to real-world geriatric cardiology. Our objective was to perform a comparative methodological analysis of two contemporary European cardiovascular RCTs, HI-COR-65 and SENEKA, as illustrative cases and to propose a geriatric-oriented framework for improving trial design in older adults with cardiovascular multimorbidity. We qualitatively compared both trials across five domains: population representativeness, clinical objectives and outcome hierarchy, exclusion criteria, multidisciplinarity, and patient perspective, including participation burden and patient-reported outcomes. Both trials represent important progress by explicitly enrolling older adults with clinically relevant cardiovascular multimorbidity. However, the analysis identified recurring limitations: geriatric complexity is acknowledged but not operationalized through comprehensive geriatric assessment or vulnerability-based stratification; safety and participation requirements may preferentially select robust older adults; outcome hierarchies remain variably aligned with patient priorities; and intervention models are predominantly pharmacological and monodisciplinary. Future cardiovascular trials in older adults should move from chronological inclusion toward pragmatic, complexity-sensitive design. We propose the Comprehensive Geriatric Cardiology (COMGERCARDIO) framework, which integrates baseline geriatric assessment, vulnerability-adaptive interventions, hierarchical patient-centered outcomes, stratified analysis, and implementation evaluation to generate evidence that is scientifically robust, clinically usable, and equitable for aging populations.

## 1. Introduction

Population aging and multimorbidity now define the clinical substrate of cardiovascular medicine. Recent epidemiological and clinical studies show that multimorbidity is common in later life and is associated with higher mortality, disability, polypharmacy, and healthcare use [1,2,3]. This has generated calls to move from fragmented, single-disease guidance toward integrated, patient-prioritized care models that account for treatment interactions, cumulative burden, and individual goals [4,5,6]. Cardiovascular disease is central to this burden: it becomes increasingly prevalent with age and frequently coexists with chronic kidney disease, anemia, frailty, cognitive impairment, sarcopenia, functional decline, and social vulnerability [7,8,9,10,11]. Acute coronary syndrome and heart failure illustrate this challenge particularly well, because older patients often present with comorbidity, frailty, renal dysfunction, polypharmacy, and difficulty achieving guideline-directed pharmacotherapy, all of which influence prognosis and treatment tolerance [12,13,14,15,16,17,18,19,20].

The problem addressed in this Perspective is the mismatch between this complex clinical reality and the simplified populations on which much cardiovascular evidence is based. The underrepresentation of older adults in randomized trials has long been recognized [21], even though randomized clinical trials (RCTs) remain essential for causal inference [22]. Older people with frailty, disability, cognitive impairment, multimorbidity, limited life expectancy, or high treatment burden are often underrepresented or indirectly excluded [23,24,25,26,27], and age-based restrictions or age-related pharmacokinetic and pharmacodynamic heterogeneity may further limit applicability [28,29]. The international multimorbidity and recent trial-methodology literature therefore emphasizes the need for pragmatic designs capable of capturing chronic complexity without sacrificing scientific rigor [30,31]. Consequently, trial participants may be chronologically old but biologically and functionally selected, leaving clinicians to extrapolate recommendations from patients who are healthier, more adherent, and easier to monitor than those encountered in everyday practice.

This evidence gap is clinically important because the success of cardiovascular therapy in older adults cannot be judged solely by disease-specific endpoints. Survival, hospitalization, and biomarker changes remain relevant, but they must be interpreted alongside functional independence, symptom burden, treatment burden, quality of life, caregiver involvement, and patient priorities. When these dimensions are absent from eligibility criteria, outcome hierarchies, and analyses, evidence-based medicine risks becoming less applicable to the population with the greatest need for evidence.

The objective of this article is to critically analyze the methodological design of two contemporary ongoing European RCTs involving older adults with cardiovascular disease: HI-COR-65 [32] and SENEKA [33]. These studies were selected because they are current, multicenter randomized trials that explicitly enroll older adults with cardiovascular multimorbidity, but they differ in clinical scenario, follow-up intensity, and outcome hierarchy. HI-COR-65 represents a post-acute coronary syndrome intervention with a patient-reported primary endpoint, whereas SENEKA represents pharmacological optimization in heart failure and chronic kidney disease. Their comparison therefore provides an illustrative, practice-oriented basis for identifying structural strengths and limitations in current geriatric cardiovascular trial design and for proposing the COMGERCARDIO framework.

## 2. Methodological Analysis of Cardiovascular Contemporary Trials in Older Adults

Explanatory trials prioritize internal validity by restricting heterogeneity. Yet heterogeneity is precisely the defining feature of older populations. This creates a methodological paradox: the more rigorous the trial, the less applicable are the results to geriatric patients [34]. Clinical consequences include overestimation of therapeutic benefits, underestimation of harm, and ambiguity in treatment protocols. In the absence of direct empirical evidence, clinicians must extrapolate findings from younger or less complex populations, transforming clinical decision-making into a task reliant on subjective discernment [9,35]. To illustrate recurring methodological patterns, we analyzed two contemporary prospective cardiovascular RCTs conducted in elderly populations. These trials were selected a priori as illustrative cases for four reasons: (i) both are ongoing European multicenter randomized trials registered in the EU Clinical Trials system; (ii) both focus on older patients with clinically relevant cardiovascular multimorbidity; (iii) they address high-burden scenarios in everyday geriatric cardiology, namely post-acute coronary syndrome (ACS) recovery and heart failure (HF)–chronic kidney disease (CKD) treatment optimization; and (iv) they represent complementary trial logics, one primarily patient-centered and one primarily guideline/process-driven. The first is HI-COR-65 (EU CT 2025-522421-36-00), which evaluates the effect of intravenous iron on the quality of life (QoL) of elderly patients with ACS [32]. The second is the SENEKA study (EU CT number 2024-513971-42-00), designed to define the optimal management strategy for HF and CKD among elderly patients [33]. Our objective is not to critique individual protocols but to identify structural tendencies in contemporary geriatric cardiovascular trial design. The evaluation is based on publicly available or reasonably inferred information without breaching clinical trial confidentiality.

HI-COR-65 is a phase IV, randomized, open-label, multicenter clinical trial assessing whether intravenous ferric carboxymaltose improves quality of life (QoL) in patients aged ≥65 years with iron deficiency following ACS. Participants are randomized 1:1 to receive a single dose of intravenous iron or standard care and are followed for 12 months. The primary outcome is changes in quality of life (EQ-5D-5L) at 6 and 12 months. Secondary outcomes include frailty, heart failure events, reinfarction, stroke, mortality, inflammatory markers, iron metabolism parameters, and exploratory biomarkers of biological aging [32]. SENEKA is a multicenter, randomized, open-label, parallel-group trial assessing whether sodium zirconium cyclosilicate (SZC) enables the optimization of renin–angiotensin–aldosterone system inhibitor (RAASi) therapy in patients ≥ 70 years with HF and CKD who have hyperkalemia or are at high risk. Participants are randomized 1:1 to SZC plus RAASi optimization or standard care and followed for 3 months. The primary endpoint is the proportion of patients achieving at least a 25% increase toward guideline-recommended RAASi target doses. Secondary and exploratory outcomes include higher-dose achievement rates, biomarker changes, renal function, QoL, hospitalizations, cardiovascular events, and safety [33]. Table 1 summarizes the characteristics and comparatives of both trials. A detailed analysis follows.

### 2.1. Comparative Methodological Critical Analysis

#### 2.1.1. Population Representativeness

A central question in both trials is whether their populations truly reflect the complexity of real-world older adults with cardiovascular multimorbidity. Although each study targets elderly patients with significant comorbid conditions (ACS with iron deficiency in one [32], and HF with CKD and hyperkalemia risk in the other [33]), multimorbidity functions mainly as a diagnostic inclusion criterion rather than as a multidimensional determinant of prognosis, treatment tolerance, adherence, and competing risks. Geriatric syndromes such as cognitive impairment, sarcopenia, disability, and polypharmacy are not systematically integrated into the design, nor is their impact on outcomes addressed. Frailty is partially acknowledged through selected outcome measures, but neither protocol incorporates a Comprehensive Geriatric Assessment framework [36] as a core selection or stratification strategy. Patients with severe instability, advanced cardiovascular disease, or limited life expectancy are excluded. Although ethically and logistically justified, this likely results in cohorts of relatively robust older adults. Institutionalized individuals and those with significant functional dependence may also be underrepresented due to the demands of protocolized follow-up, repeated visits, and treatment titration.

Consequently, despite intentionally enrolling older adults with multimorbidity, both trials may fail to capture the frailest and most complex segment of the geriatric cardiovascular population. What emerges is a selected elderly phenotype. Common exclusions (severe functional impairment, advanced comorbidity, complex disease stages, and inability to adhere strictly to protocol procedures) filter out biological and functional aging while retaining chronological age criteria. This produces a form of healthy participant bias in geriatric trials: the population most in need of evidence (i.e., the complex, dependent older patient) remains systematically underrepresented.

#### 2.1.2. Clinical Design and Objectives

A second structural issue concerns the hierarchy of outcomes embedded in both trials. Traditional cardiovascular research prioritizes biomarker modification, surrogate physiological endpoints, and disease-specific events, whereas, in geriatric populations, functional status, independence, symptom burden, and QoL often take precedence. In HI-COR-65, QoL is the primary endpoint, representing a shift toward patient-centered evaluation and aligning with geriatric priorities, where maintenance of daily function and well-being may outweigh purely biological improvements. Nonetheless, the inclusion of numerous biomarker and mechanistic secondary outcomes reflects the persistent influence of a physiology-driven framework [32]. By contrast, SENEKA defines pharmacological optimization (achievement of RAASi target doses) as its primary endpoint. While clinically relevant from a guideline and prognostic perspective, this is a process-based surrogate outcome rather than a direct measure of patient experience. Patient-reported outcomes and QoL measures are included as secondary or exploratory endpoints [33]. Therapeutic success is therefore primarily defined by dose attainment rather than preserved autonomy, symptom relief, or maintained functionality.

This distinction is particularly relevant in older adults. For an octogenarian patient, avoiding hospitalization or reaching a target drug dose may be less meaningful than preserving independence [37]. A therapy that prolongs survival but accelerates disability may be perceived as a failure from the patient’s perspective. Although both trials incorporate elements of patient-centered assessment, they illustrate the persistent tension between physiological success and clinical meaning. In geriatric care, outcome hierarchies require reconsideration, placing autonomy, function, and lived experience at the forefront rather than as downstream consequences of biomarker optimization.

#### 2.1.3. Interventions and Multidisciplinarity

Both trials evaluate pharmacological interventions largely in isolation. While clinically relevant and guideline-aligned, these strategies reflect a monodisciplinary, drug-centered model of intervention. In contrast, geriatric cardiovascular care is inherently multidisciplinary [38]. Outcomes in older adults depend not only on medication optimization but also on nutrition, physical rehabilitation, cognitive support, social resources, caregiver education, and functional monitoring. Treatment success is therefore shaped by contextual and system-level factors as much as by pharmacological efficacy. Interventions shown to be effective under controlled conditions may underperform in real-world settings if these surrounding determinants are not addressed.

Neither protocol structurally embeds interdisciplinary collaboration (such as formal integration of geriatrics, nursing, pharmacy, rehabilitation, or social care) into the intervention model. Although multimorbidity is present, care delivery remains specialty-driven rather than system-oriented. Without incorporating multidisciplinary frameworks into trial design, demonstrated efficacy may not translate into sustainable real-world effectiveness for complex older patients.

#### 2.1.4. Exclusion Criteria and Potential Bias: The Systematic Removal of Reality

Exclusion criteria represent a central methodological driver of the evidence gap in geriatric cardiovascular research. While intended to enhance internal validity and ensure safety, they often eliminate the very characteristics that define older adults with cardiovascular disease: a complex intersection of age-related physiological changes, chronic comorbidities, and specific functional limitations [1,38]. Yet these conditions are not peripheral: they constitute the clinical reality of geriatric cardiology, shaping prognosis, treatment tolerance, adherence, and patient priorities.

Both trials apply clinically understandable safety-driven exclusions [32,33]. However, cumulatively, these criteria may select patients who are chronologically older but physiologically less vulnerable. The resulting population may exhibit fewer competing risks and greater functional reserve than typical real-world patients. The evidence generated thus applies to a hypothetical individual: aged in years, but not in frailty, complexity, or trajectory. This structural filtering preserves internal validity while narrowing external applicability. In geriatric cardiovascular care, where vulnerability and multimorbidity drive therapeutic decisions, exclusion criteria may inadvertently construct a parallel reality that diverges from everyday clinical practice.

#### 2.1.5. Patient Perspective and Burden of Participation

Participation in clinical trials requires substantial logistical and cognitive resources: repeated visits, structured monitoring, strict adherence, and comprehension of informed consent. These demands presuppose functional independence, organizational capacity, and health literacy, attributes that cannot be assumed in older populations. Many older adults depend on caregivers, transportation support, and structured assistance to access healthcare. Cognitive impairment, sensory limitations, fatigue, and multimorbidity further complicate sustained participation in protocol-driven research [39]. Yet trial designs rarely integrate these contextual factors into structural planning. Participation burden is implicitly considered manageable rather than treated as a determinant of eligibility and sustainability. As a result, enrollment becomes inherently selective. Those most likely to participate are the most robust older adults with preserved cognition and stronger support networks. Frail, socially isolated, or cognitively vulnerable individuals are less likely to enroll or remain engaged. This participation bias compounds the effects of formal exclusion criteria, further narrowing external applicability. In geriatric cardiovascular research, explicitly addressing participation burden is essential to ensure that generated evidence applies to the broader population encountered in routine practice.

Table 2 summarizes the comparative analysis of both trials across the five domains above described. Overall, HI-COR-65 demonstrates a stronger patient-centered orientation through its primary QoL endpoint, although its analytic structure remains biologically framed. SENEKA follows a guideline-optimization logic centered on pharmacologic targets, with patient-centered outcomes positioned as secondary. Both trials share structural limitations typical of conventional RCTs in geriatric cardiology: complexity acknowledged but not stratified, monodisciplinary intervention models, exclusion-driven representativeness gaps, and participation burden contributing to selection bias.

## 3. Toward a New Paradigm: Pragmatic Geriatric Cardiovascular Trials

Traditional RCTs often maximize internal validity at the expense of representativeness, reinforcing the well-documented evidence gap affecting multimorbid older populations [40,41,42]. Despite bearing the highest burden of cardiovascular disease, older adults remain markedly underrepresented in RCTs, contributing to uncertainty in clinical decision-making [43]. The trials here analyzed, HI-COR-65 and SENEKA, incorporate older adults into cardiovascular research but still lack the heterogeneity, frailty, and competing risks normative in geriatric patients [32,33]. Evidence in cardiology trials including geriatric populations must reflect real-world complexity to be ethically and clinically meaningful.

To address this issue, we propose a shift from the predominantly explanatory RCT designs toward pragmatic geriatric trials. This transition does not imply reduced methodological rigor; rather, it redefines rigor in terms of maximizing the applicability and real-world effectiveness of interventions in geriatric clinical practice. We propose four foundational principles to guide this paradigm shift.

Principle 1: Patient-relevant outcomes as primary endpoints

Traditional cardiovascular research prioritizes mortality, hospitalization, and biomarker changes. Yet in older adults, functional independence, symptom burden, and QoL trajectory often outweigh longevity alone [1,44]. Comprehensive outcome models should therefore incorporate functional status (e.g., activities of daily living), treatment burden, goal concordance, and symptom control as primary or co-primary endpoints. Mortality remains important but should not dominate endpoint hierarchies when survival gains may occur at the cost of disability. This reorientation aligns therapeutic success with patient-centered care principles and broader calls within clinical research to center patient-reported outcomes and real-world health status measures [8,11,45,46]. Traditional endpoints remain valuable but should not eclipse the outcomes that determine everyday autonomy and lived experience in older adults.

Principle 2: Integrated geriatric care interventions

Conventional cardiovascular trials traditionally evaluate isolated pharmacological agents, whereas geriatric care is delivered through coordinated, multidisciplinary systems. Evidence indicates that co-managed approaches improve outcomes in older adults with complex conditions [47,48,49]. Future trials should therefore assess integrated strategies, such as pharmacotherapy combined with rehabilitation, deprescribing protocols, or cardiology–geriatrics co-management, rather than single interventions detached from context. Testing care systems rather than isolated treatments enhances external validity and better reflects clinical reality. Studies in other fields, including Comprehensive Geriatric Assessment combined with multidisciplinary interventions, have shown improved patient-centered outcomes when multidimensional care is assessed as a unified strategy [47,50]. Cardiovascular research should adopt similar system-level designs.

Principle 3: Complexity stratification instead of complexity exclusion

Frailty, polypharmacy, renal dysfunction, cognitive impairment, and high competing mortality risk have historically justified the exclusion of older adults from RCTs [20,51,52,53]. Yet these features define geriatric cardiovascular practice rather than distorting it. Instead of excluding complexity to preserve homogeneity, trials should stratify participants according to vulnerability levels (robust, pre-frail, and frail) using validated instruments such as the Frailty Phenotype [54] or the Clinical Frailty Scale [55]. Analyzing treatment effects across vulnerability strata, transforms heterogeneity from a statistical nuisance into clinically actionable knowledge. Structured frailty assessments are increasingly recognized as modifiers of risk and prognosis in older adults with cardiovascular disease [56]. Incorporating stratified analyses allows evidence to inform individualized decision-making rather than applying averaged effects across biologically diverse populations.

Principle 4: Mixed quantitative–qualitative methodology

Quantitative endpoints alone cannot fully capture therapeutic success in vulnerable populations. Mixed-methods research, including embedded qualitative substudies exploring patient experience, caregiver burden, acceptability, and feasibility, has been recommended for evaluating complex interventions [57,58,59,60]. Integrating qualitative components within pragmatic trials clarifies why interventions succeed or fail in real-world contexts and illuminates trade-offs invisible to purely numerical analyses. Understanding treatment effectiveness in older adults requires both measurable outcomes and insight into lived experience. By examining patient and caregiver perspectives alongside statistical effects, mixed-method designs enhance interpretability and humanize trial results, particularly when statistical benefit does not align with perceived clinical value.

In summary, transitioning to pragmatic geriatric trials strengthens scientific standards by aligning methodological rigor with clinical reality. Stratifying rather than excluding complexity, prioritizing outcomes meaningful to older patients, evaluating integrated care models, and incorporating qualitative inquiry can generate evidence that is both internally valid and externally applicable. In aging societies characterized by multimorbidity, the ethical imperative of research extends beyond precision to representativeness.

### The COMGERCARDIO Framework

**Figure 1:**
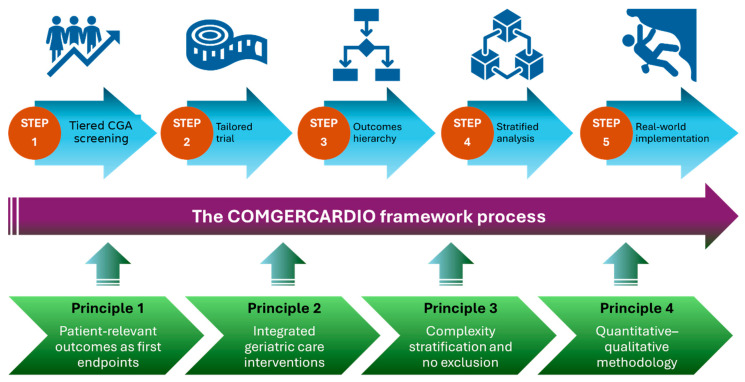
The COMGERCARDIO framework. Conceptual representation of the Comprehensive Geriatric Cardiology (COMGERCARDIO) framework proposed to guide the design of pragmatic cardiovascular randomized clinical trials in older adults with multimorbidity. The model integrates four foundational principles (patient-centered outcomes, integrated geriatric care interventions, complexity stratification, and mixed quantitative–qualitative methodology) into a five-step process: baseline complexity assessment using CGA or tiered CGA-informed screening, vulnerability-adaptive intervention strategies, hierarchical outcome prioritization, stratified analysis by frailty and multimorbidity, and real-world implementation assessment. CGA, comprehensive geriatric assessment.

Before describing the framework, its central principle should be made explicit: in geriatric cardiovascular research, methodological rigor should be measured not only by control of heterogeneity but also by the capacity to generate evidence applicable to heterogeneous patients. COMGERCARDIO is therefore designed to convert geriatric complexity—frailty, function, cognition, multimorbidity, polypharmacy, social support, and treatment burden—from an exclusionary obstacle into a structured design and analytic variable. Its goal is to help investigators design pragmatic cardiovascular RCTs that preserve internal validity while improving external validity, equity, and clinical usability. To operationalize these principles, we propose a structured model: the Comprehensive Geriatric Cardiology framework (COMGERCARDIO). Rather than modifying isolated aspects of trial design, COMGERCARDIO offers a stepwise architecture that integrates geriatric principles into cardiovascular research. A diagram of this framework process is graphically depicted in Figure 1, including the above principles in which the framework is based upon.

Step 1: Baseline complexity assessment

Participants should undergo baseline characterization of geriatric complexity, ideally through a structured Comprehensive Geriatric Assessment (CGA) including frailty status, cognitive function, physical performance, functional independence, comorbidity burden, polypharmacy, and social context. However, COMGERCARDIO can be operationalized pragmatically through a tiered assessment strategy in large multicenter trials. Brief validated tools such as the Clinical Frailty Scale [55], gait speed, activities-of-daily-living assessment, medication review, and short cognitive screening may be used at prescreening or baseline to identify vulnerability without imposing excessive time or cost. Full or abbreviated CGA modules can then be targeted to participants above predefined vulnerability thresholds or to representative subcohorts. Standardized training, electronic case-report forms, and central quality control would preserve harmonization across sites while limiting recruitment barriers. Complexity is therefore not treated as a confounder but as a defining clinical variable, ensuring that vulnerability is systematically characterized rather than implicitly filtered.

Step 2: Adaptive intervention

Therapeutic intensity is tailored according to vulnerability level. Robust patients may receive full guideline-directed optimization, whereas pre-frail and frail individuals may undergo calibrated titration strategies, deprescribing safeguards, or co-managed care models. To preserve methodological rigor, adaptive rules should be prespecified before randomization, linked to reproducible vulnerability strata, and implemented through standardized titration algorithms, safety thresholds, and blinded outcome adjudication whenever possible. In strictly blinded phase III regulatory trials, COMGERCARDIO may be incorporated more conservatively by using geriatric assessment for stratified randomization, predefined subgroup analyses, safety monitoring, and patient-centered outcomes while keeping the investigational treatment standardized. By contrast, the full vulnerability-adaptive intervention model is particularly suited to phase IV, pragmatic effectiveness, and implementation trials, where the objective is to evaluate clinically usable treatment strategies under real-world conditions. This adaptive approach replaces the binary logic of “eligible versus excluded” with vulnerability-informed personalization that mirrors clinical practice.

Step 3: Hierarchical outcomes

The framework proposes a restructured outcome hierarchy:

1. Primary outcomes: patient-centered measures such as functional independence, symptom burden, QoL trajectory, goal concordance, and treatment burden.
2. Secondary outcomes: clinical events including mortality, hospitalization, and major cardiovascular events.
3. Exploratory outcomes: biological and surrogate markers.

This hierarchy acknowledges that, in older adults, clinical meaning precedes physiological optimization. Mortality and biomarkers remain important but do not alone define therapeutic success.

Step 4: Stratified analysis

Treatment effects are analyzed according to frailty status and multimorbidity burden. Rather than averaging outcomes across heterogeneous populations, the framework explicitly evaluates effect modification by vulnerability level. Heterogeneity thus becomes a source of clinically actionable insight, supporting individualized care.

Step 5: Real-world implementation analysis

COMGERCARDIO incorporates an implementation layer assessing feasibility, acceptability, adherence sustainability, caregiver burden, and system-level applicability. Participation burden and contextual determinants are treated as measurable outcomes rather than incidental considerations. This ensures that efficacy observed under study conditions can translate into real-world effectiveness.

In summary, the COMGERCARDIO framework bridges the gap between cardiovascular trials focused on isolated treatments and geriatric medicine, which delivers multidimensional systems of care. By embedding complexity assessment, adaptive intervention, patient-centered outcome hierarchies, stratified analysis, and implementation evaluation within a unified structure, the model seeks to produce evidence that is both internally rigorous and externally relevant. In aging societies marked by multimorbidity and vulnerability, evidence must not only be precise, but it must also be usable. COMGERCARDIO aims to achieve both.

**Figure 2:**
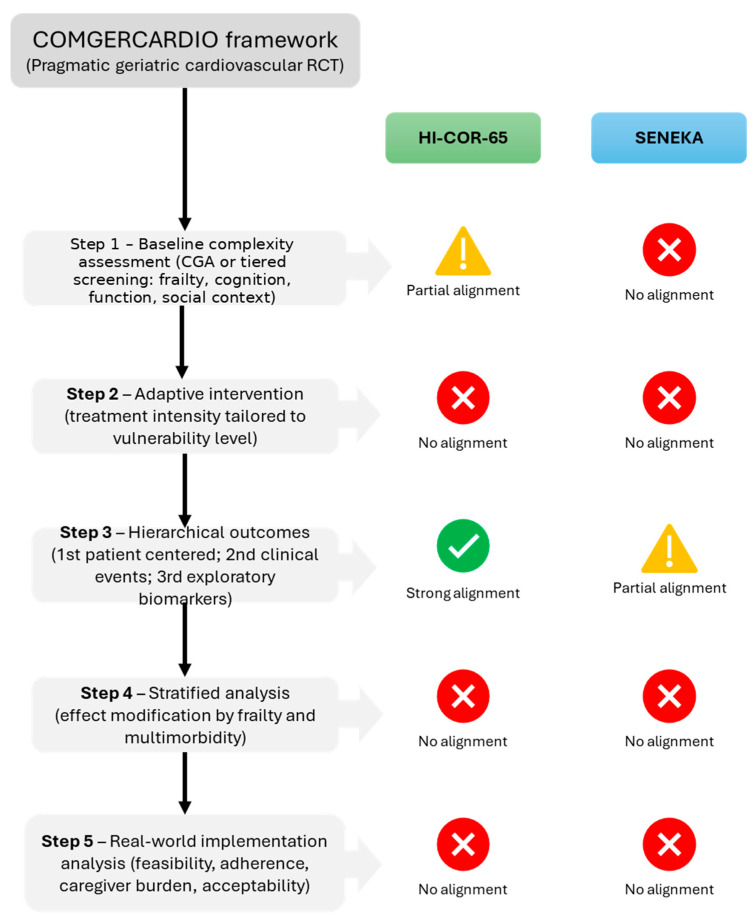
Alignment of HI-COR-65 and SENEKA with the COMGERCARDIO framework. The figure operationalizes the five COMGERCARDIO steps as a visual appraisal matrix. Each row corresponds to one framework step: (1) baseline complexity assessment through CGA or validated tiered screening, including frailty, cognition, function, and social context; (2) vulnerability-adaptive intervention; (3) hierarchical outcomes prioritizing patient-centered measures, followed by clinical events and then biomarkers; (4) stratified effect analysis by frailty and multimorbidity; and (5) real-world implementation analysis, including feasibility, adherence, caregiver burden, and acceptability. The two columns compare HI-COR-65 and SENEKA. Green check marks indicate strong alignment, yellow warning signs indicate partial alignment, and red cross marks indicate no formal alignment. HI-COR-65 shows strong alignment for the patient-centered outcome hierarchy and partial alignment for baseline complexity assessment, whereas SENEKA shows partial alignment for outcome hierarchy. Neither study formally incorporates vulnerability-adaptive interventions, stratified analysis, or implementation evaluation. CGA, comprehensive geriatric assessment; RCT, randomized clinical trial.

Table 3 and Figure 2 summarize how the two RCTs analyzed in this work would align with each step of the proposed COMGERCARDIO model. Overall, HI-COR-65 aligns more closely with its principles due to its patient-centered primary endpoints but lacks structured complexity, stratification, and adaptive design. SENEKA reflects a conventional explanatory RCT structure centered on pharmacologic optimization, with limited patient-centered prioritization. Neither trial operationalizes vulnerability as an analytic dimension nor integrates multidisciplinary care or implementation of science components.

## 4. Discussion

This comparative methodological analysis highlights a persistent structural tension in cardiovascular research involving older adults. Although contemporary trials increasingly include elderly patients chronologically, they often fail to fully integrate the biological, functional, and social complexity that defines geriatric clinical reality [61]. The two analyzed protocols illustrate meaningful progress, yet they also reveal enduring limitations related to population representativeness, outcome hierarchies, intervention models, exclusion criteria, and participation burden.

A central implication from our study is that the cardiovascular evidence base for older adults continues to be built around a physiologically optimized but clinically simplified model of aging. While geriatric conditions such as frailty, multimorbidity, cognitive vulnerability and competing risks are often acknowledged conceptually, they are rarely operationalized in trial design, as observed in the HI-COR-65 and SENEKA trials. Previous work has already demonstrated that older adults with multimorbidity remain systematically underrepresented in randomized trials [41,52]. When these characteristics are excluded or indirectly filtered through demanding participation requirements, trial populations may reflect chronological age without capturing the multidimensional complexity of geriatric patients.

Heterogeneity is a defining feature of aging populations rather than a methodological limitation. Older adults exhibit wide variation in functional reserve, comorbidity burden, cognitive status, and social support, all of which may modify both treatment benefits and harms [8,42,62]. Trial designs that attempt to minimize this variability in pursuit of homogeneity risk producing evidence that is internally valid but poorly applicable to real-world clinical practice. Consequently, clinical recommendations derived from selected trial populations may generate two opposing patterns of care in complex older adults: overtreatment driven by strict adherence to protocolized therapeutic targets, and undertreatment resulting from clinician uncertainty when evidence appears inapplicable to vulnerable patients [5,41,42,52]. Both phenomena stem from the same structural gap: an evidence base that insufficiently represents geriatric complexity.

Outcome selection represents another key limitation in current cardiovascular research involving older adults. Traditional trials prioritize endpoints such as mortality, hospitalization, or biomarker changes. Although these outcomes remain clinically important, they do not always align with the priorities of older patients, for whom maintaining functional independence, reducing symptom burden, and preserving quality of life may be more meaningful than survival alone [8,45]. When these dimensions are not incorporated into primary outcome hierarchies, guideline recommendations risk being derived from endpoints that lack universal relevance for the populations they intend to serve [61]. This limitation also has implications for health policy decisions, as pragmatic trials are uniquely positioned to inform policymakers and healthcare providers about the real-world effectiveness and cost implications of treatments [63].

A further gap emerges from the mismatch between the intervention models studied in trials and the way care is delivered in geriatric practice. Cardiovascular randomized trials typically evaluate isolated pharmacological interventions, as exemplified by the SENEKA trial. In contrast, geriatric care is inherently multidisciplinary, integrating medication optimization, rehabilitation strategies, nutritional support, cognitive management, and social resources. Evidence suggests that comprehensive geriatric assessment and multidisciplinary care models improve outcomes in older adults [47]. However, these integrated approaches are rarely embedded within the architecture of cardiovascular trials, limiting their external validity and translational relevance.

Participation burden also represents an important but often overlooked determinant of representativeness. Repeated visits, complex monitoring requirements, and rigid study protocols may disproportionately exclude frail individuals or those with limited functional or social resources. Consequently, trial populations may become selectively enriched with relatively robust older adults even in the absence of explicit exclusion criteria. Recognizing participation burden as a methodological factor is therefore essential to improving the representativeness of cardiovascular trials involving older populations. Incorporating implementation science principles and mixed-method evaluations (including qualitative assessments of feasibility, caregiver burden, and acceptability) may improve the interpretability and sustainability of research findings [31,57,64].

Addressing these challenges requires more than incremental adaptation; it demands a structural redesign of research methodology. Several methodological shifts are necessary. First, complexity should be stratified rather than excluded. Frailty assessment and comprehensive geriatric evaluation can be incorporated into baseline characterization and subgroup analyses, transforming vulnerability from a confounder into a clinically informative modifier [54,57,65,66]. Second, outcome hierarchies should be recalibrated to prioritize patient-centered endpoints such as functional independence, quality-of-life trajectories, symptom burden, and treatment burden. This approach aligns with broader calls for goal-oriented and patient-prioritized care in multimorbidity [8,26,31,67]. Third, trial designs should move beyond isolated therapeutic strategies toward the evaluation of integrated interventions that better reflect routine clinical practice. Pragmatic methodological frameworks, such as PRECIS-2, provide guidance for designing trials with greater real-world applicability [68].

Within this context, the COMGERCARDIO framework offers a conceptual model for integrating geriatric principles into cardiovascular research. The framework proposes the systematic incorporation of baseline complexity stratification rather than exclusion, adaptive intervention strategies tailored to vulnerability, hierarchical outcome structures prioritizing patient-centered endpoints, stratified analyses by frailty and multimorbidity, and formal evaluation of feasibility and implementation in real-world settings. Embedding these elements into trial design may allow cardiovascular research to generate evidence that is both methodologically robust and clinically actionable.

The adoption of vulnerability-stratified research models such as COMGERCARDIO could also influence clinical practice and guideline development. Evidence generated from stratified analyses would allow clinicians to contextualize treatment decisions rather than extrapolate results from homogeneous trial populations. Identifying differential treatment effects across frailty strata, for example, could inform individualized risk–benefit discussions and deprescribing strategies. At the guideline level, data from pragmatic and vulnerability-sensitive trials could support more nuanced recommendations, moving beyond uniform dose targets or binary treatment indications toward conditional guidance based on functional reserve, multimorbidity burden, or patient priorities [69,70,71]. Integrating stratified evidence into guideline development would better support shared decision-making, deprescribing when appropriate, and adaptive treatment intensity aligned with patient goals [45,69]. Such evolution could help resolve the tension clinicians often experience between strict adherence to guideline algorithms and individual patient care.

The systematic underrepresentation of complex older adults in clinical trials raises important ethical considerations. When frail, cognitively impaired, or multimorbid individuals are excluded, those most exposed to therapeutic risks become the least represented in the evidence base [52,72,73]. This imbalance creates a structural inequity within evidence-based medicine. Older adults with multimorbidity bear the greatest burden of cardiovascular disease and polypharmacy, yet they frequently receive treatments supported by data derived from healthier populations. This asymmetry challenges the ethical principles of justice and beneficence that underpin clinical research [41,42]. Ensuring that vulnerable populations are meaningfully represented, stratified, and analyzed in clinical trials is therefore not only a methodological improvement but also an ethical imperative.

Taken together, these considerations highlight the need for a paradigm shift in cardiovascular research involving older adults. Integrating geriatric complexity into trial design is essential if evidence generation is to remain relevant in aging societies. Frameworks such as COMGERCARDIO provide a structured pathway for achieving this transformation, bridging the gap between methodological rigor and clinical applicability.

An important clinical utility of COMGERCARDIO is that it can serve as a trial-design checklist and implementation bridge for high-complexity cardiovascular pathways. Post-surgical cardiovascular care illustrates this need. Remote monitoring systems after cardiac surgery can capture blood pressure, heart rate, oxygen saturation, temperature, glucose, ECG, symptoms, wound status, medication adherence, and patient-reported recovery data; however, the value of these data depends on translating heterogeneous streams into actionable risk stratification. Recent studies show that remote patient monitoring after cardiac surgery is feasible and can support early detection of complications, while machine-learning clinical decision support systems can help allocate limited monitoring resources to patients at greater post-discharge risk [74,75]. In older adults, such clinical decision support systems should be evaluated not only for discrimination and calibration, but also for effects on workload, equity, patient burden, caregiver burden, readmissions, functional recovery, and clinician trust.

Retrieval-augmented generation may further strengthen post-surgical cardiovascular monitoring by linking patient-specific data to curated, up-to-date clinical knowledge and institutional pathways. Early surgical retrieval-augmented generation systems have shown that grounding large language model outputs in guideline-based or physician-verified knowledge bases can improve relevance and reduce hallucination risk compared with unguided generative models [76,77]. For post-surgical cardiovascular care, a retrieval-augmented generation-enabled, human-in-the-loop clinical decision support systems could synthesize telemetry, laboratory results, medication titration, wound-status reports, rehabilitation data, and recent evidence to generate explainable monitoring recommendations. Such tools require prospective validation, auditability, data governance, safeguards for urgent escalation, and clear clinician accountability before being incorporated into trials or routine care.

Psychological and neuropsychiatric morbidity should also be treated as part of cardiovascular complexity rather than as a peripheral comorbidity. Delirium and postoperative cognitive dysfunction are common after cardiac surgery and are associated with inflammation, baseline vulnerability, functional decline, prolonged hospitalization, and later neuropsychiatric symptoms [78,79]. Depression is frequently underdiagnosed in cardiac surgery and has been linked to worse recovery, readmissions, and mortality, while patients receiving advanced cardiac therapies such as left ventricular assist devices may experience depression, existential distress, loss of autonomy, and suicidality [78,80,81]. These observations reinforce the need for trial designs that include baseline psychological screening, delirium prevention and monitoring, caregiver assessment, and patient-centered endpoints capturing mental health and social support.

Finally, future cardiovascular trial methodology must also account for the organizational and environmental context of advanced care. Cardiothoracic surgery is both technologically intensive and resource intensive, making it an appropriate field for “twin transformation”, the deliberate alignment of digital innovation with environmental sustainability [82]. Digital tools, remote follow-up, interoperable records, AI-based risk stratification, and simulation may improve precision and reduce unnecessary visits, tests, waste, and resource use when coupled with sustainable institutional practices. At the same time, patient-centered cardiac surgery emphasizes psychological preparation, shared decision-making, social support, rehabilitation, symptom management, and quality-of-life recovery as determinants of surgical outcomes [83]. These domains are directly compatible with COMGERCARDIO because they expand trial success beyond technical efficacy toward lived recovery, implementation feasibility, and value-sensitive care.

## 5. Conclusions

Contemporary cardiovascular research is increasingly conducted in an aging population, yet its methodological foundations remain largely rooted in assumptions of biological uniformity. Although trials such as HI-COR-65 and SENEKA increasingly enroll elderly participants, important limitations persist, including incomplete representation of geriatric complexity, outcome hierarchies dominated by physiological endpoints, monodisciplinary intervention models applied to multidimensional patients, and participation burdens that preferentially recruit the most robust older adults. As longevity increases and multimorbidity becomes the dominant clinical scenario, the central challenge in geriatric cardiology is no longer solely therapeutic innovation but the generation of evidence applicable to the heterogeneous population encountered in daily practice. Randomized trials remain essential for advancing care, but their design must evolve to incorporate the complexity that characterizes older patients.

The COMGERCARDIO framework provides a conceptual roadmap for this transition. By integrating vulnerability stratification, adaptive intervention strategies, hierarchical patient-centered outcomes, stratified analyses by frailty and multimorbidity, and formal evaluation of implementation and feasibility, the model redefines methodological rigor as the ability to generate evidence that is both scientifically sound and clinically applicable. Without such methodological evolution, traditional explanatory trial models risk perpetuating an evidence gap for the populations most affected by cardiovascular disease. Aligning research design with geriatric clinical reality will be essential if clinical guidelines, therapeutic decisions, and shared care planning are to be grounded in evidence that truly reflects the needs and priorities of aging populations.

## 6. Limitations, Future Directions, and Implementation Perspectives of the COMGERCARDIO Framework

Beyond its conceptual contribution, COMGERCARDIO has potential clinical utility at four levels: (1) as a protocol-design checklist to ensure that frailty, cognition, function, multimorbidity, social context, and treatment burden are measured rather than ignored; (2) as an appraisal tool to judge whether trial populations and outcomes are applicable to real-world older adults; (3) as an analytic strategy for identifying vulnerability-specific treatment effects; and (4) as a translational bridge between trial results, guideline development, shared decision-making, and deprescribing or intensity-adjustment strategies. Future directions include feasibility studies of Comprehensive Geriatric Assessment integration into trial screening, pilot pragmatic trials testing adaptive interventions, development of core outcome sets for geriatric cardiovascular trials, embedding qualitative substudies, and prospective validation of whether COMGERCARDIO-informed designs improve representativeness and clinical utility.

This perspective has several limitations that should be acknowledged. First, the analysis is conceptual and interpretative in nature, based on a comparative methodological examination of contemporary cardiovascular RCTs rather than a systematic review or meta-analysis of the literature. Accordingly, the conclusions should be interpreted as illustrative rather than exhaustive representations of current trial design practices in geriatric cardiology. Second, the selected trials were evaluated through a qualitative methodological lens focusing on eligibility criteria, outcome hierarchies, and integration of geriatric principles. While this approach helps identify structural gaps between conventional cardiovascular trial methodology and the clinical complexity of older patients with multimorbidity and frailty, it inevitably involves some interpretative judgment. Third, the COMGERCARDIO framework proposed here is a conceptual model derived from the integration of principles from Comprehensive Geriatric Assessment, frailty research, and patient-centered outcomes methodology. While these domains are supported by substantial empirical evidence regarding their prognostic and clinical relevance in older adults [44,53,72], the integrated framework itself has not yet been empirically validated in prospective cardiovascular trials. Finally, the operational implications of incorporating multidimensional geriatric assessments, vulnerability-based stratification, and patient-centered outcome hierarchies into RCTs may vary across healthcare systems, regulatory environments, and research infrastructures, influencing feasibility and scalability.

COMGERCARDIO should therefore be understood as a conceptual proposal intended to stimulate methodological innovation in cardiovascular research involving older adults with multimorbidity. The model integrates established principles from Comprehensive Geriatric Assessment, frailty phenotyping, pragmatic clinical trial design, and patient-centered outcomes research into a unified structure aimed at aligning clinical trials with geriatric cardiology practice. Its conceptual foundations are supported by evidence showing that frailty and multidimensional vulnerability strongly influence prognosis, treatment tolerance, and functional outcomes in older patients with cardiovascular disease [20,72]. In addition, the methodological literature emphasizes the importance of pragmatic designs and outcomes relevant to patient functioning and quality of life in studies involving complex aging populations [68,70]. Future validation of the COMGERCARDIO model could follow a stepwise research agenda. Initial feasibility studies could evaluate the practicality of integrating structured Comprehensive Geriatric Assessment components into cardiovascular trial screening procedures, evaluating participant burden, recruitment feasibility, and data completeness. Subsequently, pilot trials could incorporate selected components of the framework (i.e., vulnerability-based stratification or hierarchical patient-centered outcomes) to explore their influence on treatment-effect heterogeneity and clinical interpretability. Later phases may involve pragmatic randomized trials or embedded methodological studies comparing conventional cardiovascular trial designs with COMGERCARDIO-informed approaches to determine whether the framework improves external validity, patient-centered relevance, and translational applicability without compromising methodological rigor.

Implementation may involve methodological and practical trade-offs. Multidimensional geriatric assessment and patient-centered outcomes can increase trial complexity and operational demands, as Comprehensive Geriatric Assessment requires evaluation of functional status, cognition, comorbidity, nutrition, and social support, often involving multidisciplinary expertise [47]. Integrating these components into cardiovascular trial protocols may require additional training, longer baseline assessments, and expanded data management. Additionally, cardiovascular trials have traditionally prioritized hard clinical endpoints such as mortality or major adverse cardiovascular events. While these outcomes remain essential, evidence indicates that functional independence, symptom burden, and quality of life are highly meaningful outcomes for older patients with cardiovascular disease [70,73]. Expanding outcome hierarchies to incorporate these domains may require broader consensus among investigators, regulators, and funding agencies. Stratifying participants by frailty or multimorbidity may also introduce statistical and analytical challenges, since increased heterogeneity can complicate power calculations and subgroup analyses. However, such heterogeneity may reveal clinically relevant insights into treatment-effect modification that are often masked in traditional trials [9,20]. Operational feasibility may represent another potential barrier, as structured geriatric assessments and multidisciplinary interventions may be more feasible in centers with established geriatric expertise than in resource-limited research environments. Finally, there is an inherent methodological tension between experimental control and real-world representativeness. While complexity-sensitive trial designs may reduce experimental simplification, they may ultimately improve clinical applicability and translational value of cardiovascular research in aging populations.

To reduce these barriers, COMGERCARDIO does not require every trial to implement a lengthy full CGA for all screened patients. A pragmatic implementation could use a short standardized geriatric screening battery at prescreening, followed by abbreviated or full CGA only in vulnerable patients, in participants crossing predefined thresholds, or in representative subgroups. This tiered model could minimize assessment time, cost, and recruitment burden while retaining the ability to describe and analyze vulnerability.

Similarly, the adaptive intervention component should be matched to trial purpose. In phase III trials intended to support regulatory approval, strict blinding and standardization may limit vulnerability-dependent tailoring; in that setting, geriatric assessment can still inform eligibility safeguards, stratified randomization, prespecified subgroup analyses, safety monitoring, and patient-centered outcome evaluation. The full adaptive strategy is more naturally aligned with phase IV pragmatic effectiveness trials and implementation studies, where treatment individualization, feasibility, adherence, and patient-centered outcomes are part of the intervention being tested.

## Figures and Tables

**Table 1:** Methodological comparison of the HI-COR-65 and SENEKA trials.

Domain	HI-COR-65	SENEKA	Comparative Insight
Clinical context	Post–ACS with iron deficiency in ≥65 years	HF + CKD + hyperkalemia risk in ≥70 years	Both focus on elderly cardiovascular populations with multimorbidity
Type of trial	Phase IV, randomized, open-label, multicenter	Randomized, open-label, multicenter, parallel-group	Similar conventional RCT structure
Primary objective	Improvement in quality of life (EQ-5D-5L)	≥25% increase toward guideline-recommended RAASi target dose	HI-COR-65 prioritizes patient-centered outcome; SENEKA prioritizes process-based pharmacologic optimization
Outcome hierarchy	QoL as primary; clinical events and biomarkers secondary	Dose optimization primary; QoL and biomarkers secondary/exploratory	Reflects physiological vs. patient-centered hierarchy tension
Population complexity addressed	Iron deficiency + ACS; limited structured frailty stratification	HF + CKD + hyperkalemia risk; no formal frailty stratification	Multimorbidity present but not operationalized through CGA in either
Frailty assessment	Includes frailty scale as secondary measure	No structured frailty-based stratification	Frailty acknowledged but not embedded in design
Exclusion impact	Excludes severe instability, advanced HF, severe anemia	Excludes severe hyperkalemia, dialysis, advanced instability	Both may systematically exclude the most vulnerable
Intervention model	Single pharmacological intervention (iron)	Potassium binder enabling RAASi optimization	Monodisciplinary pharmacologic focus in both
Multidisciplinary integration	Not structurally embedded	Not structurally embedded	Care complexity not reflected in intervention architecture
Follow-up duration	12 months	3 months	HI-COR-65 captures longer-term outcomes
Patient-reported outcomes	Primary endpoint	Secondary/exploratory	Greater patient-centered emphasis in HI-COR-65
Participation burden	Scheduled visits at baseline, 6, 12 months	Frequent visits and titration over 90 days	SENEKA potentially higher short-term monitoring burden
Potential bias	Selection toward relatively robust older adults	Similar selection toward stable, adherent participants	Participation and exclusion bias in both
External validity	Moderate; patient-centered but still structured	Moderate–limited; process-driven primary endpoint	Both internally valid but partially constrained externally
Key comparative themes			
Shared strengths			
Inclusion of elderly populations Randomized, prospective design Clinically relevant therapeutic questions			
Shared limitations			
Lack of formal geriatric complexity stratification Exclusion of the most vulnerable patients Predominantly pharmacological intervention models Limited integration of multidisciplinary systems of care			
Key differences			
HI-COR-65 adopts a patient-centered primary endpoint (quality of life) SENEKA prioritizes pharmacological optimization as primary success measure			

ACS, acute coronary syndrome; CGA, comprehensive geriatric assessment; CKD, chronic kidney disease; EQ-5D-5L, EuroQol 5-Dimension 5-Level; HF, heart failure; QoL, quality of life; RAASi, renin–angiotensin–aldosterone system inhibitor.

**Table 2:** Domain-based methodological comparison of HI-COR-65 and SENEKA trials.

Domain	HI-COR-65	SENEKA	Critical Comparative Insight
a. Population			
Population representativeness			
	Includes ≥65 years post-ACS with iron deficiency; frailty measured but not structurally integrated	Includes ≥70 years with HF + CKD + hyperkalemia risk; no formal frailty stratification	Both include elderly multimorbid patients, but complexity is diagnosis-driven rather than operationalized through structured geriatric assessment
Multimorbidity integration			
	Acknowledged but not analyzed as interacting determinant	Acknowledged through HF + CKD coexistence but not multidimensionally assessed	Multimorbidity present in inclusion criteria, not embedded as analytic framework
Frail and/or institutionalized patients			
	Likely underrepresented due to stability requirements	Likely underrepresented due to monitoring and titration intensity	Structural selection toward relatively robust older adults
b. Design & Objectives			
Clinical design and objectives			
	Primary endpoint: QoL (EQ-5D-5L)	Primary endpoint: ≥25% RAASi dose optimization	HI-COR-65 aligns more with patient-centered priorities; SENEKA prioritizes physiological/process endpoint
Outcome hierarchy			
	Patient-centered outcome primary; biomarkers secondary	Process-based pharmacologic target primary	Illustrates tension between clinical meaning vs. physiological success
c. Interventions & Multidisciplinarity			
Interventions and multidisciplinary approach			
	Single pharmacologic intervention (IV iron)	Pharmacologic intervention (SZC enabling RAASi titration)	Both evaluate isolated treatments rather than integrated care systems
Interdisciplinary integration			
	Not formally embedded	Not formally embedded	Reflect monodisciplinary cardiovascular model despite multidimensional patients
d. Exclusion Criteria & Bias			
Exclusion criteria and potential bias			
	Excludes severe instability, advanced HF, severe anemia	Excludes severe hyperkalemia, dialysis, advanced instability	Both apply safety-based exclusions that may systematically remove highest-risk patients
Complexity exclusion risk			
	Frailty and severe vulnerability indirectly filtered	High-risk renal and unstable patients excluded	Evidence may apply to “older in age but not in vulnerability” population
e. Patient Perspective			
Patient perspective & intervention burden			
	Moderate visit burden (baseline, 6, 12 months)	Intensive short-term monitoring and titration (90 days)	Participation demands favor robust, organized, caregiver-supported patients
Participation bias risk			
	Possible	Higher due to titration intensity	Trial participation itself becomes selective

ACS, acute coronary syndrome; CKD, chronic kidney disease; EQ-5D-5L, EuroQol 5-Dimension 5-Level; HF, heart failure; QoL, quality of life; RAASi, renin–angiotensin–aldosterone system inhibitor; SZC, Sodium zirconium cyclosilicate.

**Table 3:** Alignment of HI-COR-65 and SENEKA with the five steps of the COMGERCARDIO framework.

COMGERCARDIO Step	HI-COR-65	SENEKA	Framework Alignment
**Step 1—Baseline complexity assessment**			
Tiered CGA-informed assessment: frailty, cognition, function, social context	Frailty scale included as secondary measure; no structured CGA; no cognitive or social assessment embedded	No formal frailty stratification; no structured CGA; primarily disease-based inclusion	Neither protocol systematically integrates CGA or equivalent tiered screening. Complexity is diagnosis-based rather than multidimensional
Complexity stratification: robust/pre-frail/frail subgroup analysis	No predefined stratified analysis by vulnerability level	No predefined vulnerability-based stratification	Heterogeneity not operationalized; complexity partially acknowledged but not analytically leveraged
**Step 2—Adaptive intervention**			
Treatment intensity adapted to vulnerability	Single-dose IV iron regardless of frailty status	RAASi titration protocol standardized; not vulnerability-adaptive	Interventions are protocol-driven rather than vulnerability-tailored
**Step 3—Hierarchical outcomes**			
Primary: patient-centered; Secondary: clinical events; Exploratory: biomarkers	Primary endpoint: Quality of Life (EQ-5D-5L). Secondary: clinical events & biomarkers	Primary endpoint: RAASi dose optimization. Secondary: PROs, clinical events, biomarkers	HI-COR-65: Partial alignment. SENEKA: Outcome hierarchy remains physiology-driven
**Step 4—Stratified effect analysis**			
Effect modification by frailty/multimorbidity	No prespecified analysis by frailty or multimorbidity burden	No stratified analysis by vulnerability level	Average treatment effect emphasized over vulnerability-specific response
**Step 5—Real-world implementation analysis**			
Feasibility, adherence, acceptability, caregiver burden	No formal implementation analysis; moderate visit burden	No implementation science component; higher short-term monitoring burden	Feasibility and participation burden not structurally assessed
Participation burden consideration	Moderate follow-up (baseline, 6, 12 months)	Frequent titration visits over 90 days	Potential participation bias in both; greater short-term burden in SENEKA
Integrated care model multidisciplinary intervention	Pharmacologic intervention only	Pharmacologic intervention only	Monodisciplinary design; no embedded geriatric co-management
Guideline applicability potential	Moderate: patient-centered primary endpoint improves relevance	Limited: primary outcome process-based	Both internally valid; limited vulnerability-adjusted applicability

CGA, comprehensive geriatric assessment; EQ-5D-5L, EuroQol 5-Dimension 5-Level; RAASi, renin–angiotensin–aldosterone system inhibitor.

## Data Availability

No new data were created or analyzed in this study.
